# Anemia and Mineral Bone Disorder in Kidney Disease Patients: The Role of FGF-23 and Other Related Factors

**DOI:** 10.3390/ijms252312838

**Published:** 2024-11-29

**Authors:** Nazareno Carullo, David Sorbo, Teresa Faga, Sara Pugliese, Maria Teresa Zicarelli, Davide Costa, Nicola Ielapi, Yuri Battaglia, Antonio Pisani, Giuseppe Coppolino, Davide Bolignano, Ashour Michael, Raffaele Serra, Michele Andreucci

**Affiliations:** 1 “G. Jazzolino” Hospital, A.S.P. Vibo Valentia, I89900 Vibo Valentia, Italy; nazareno.carullo@gmail.com; 2 San Bortolo Hospital, ULSS 8 Berica, I36100 Vicenza, Italy; david.sorbo@aulss8.veneto.it; 3 Department of Health Sciences, “Magna Graecia” University, I88100 Catanzaro, Italy; teresa_faga@yahoo.it (T.F.); pugliesesara651@gmail.com (S.P.); gcoppolino@unicz.it (G.C.); ashourmichael@yahoo.com (A.M.); 4 Amantea Outpatient Clinic, A.S.P. Cosenza, I87032 Amantea, Italy; mteresa.zicarelli@gmail.com; 5 Department of Medical and Surgical Sciences, “Magna Graecia” University, I88100 Catanzaro, Italy; davide.costa@unicz.it (D.C.); dbolignano@unicz.it (D.B.); 6 Interuniversity Center of Phlebolymphology (CIFL), “Magna Graecia” University, I88100 Catanzaro, Italy; nicola.ielapi@uniroma1.it; 7 Department of Public Health and Infectious Disease, “Sapienza” University of Rome, I00185 Rome, Italy; 8 Department of Medicine, University of Verona, I37129 Verona, Italy; yuri.battaglia@univr.it; 9 Department of Public Health, University of Naples Federico II, I80131 Naples, Italy; antonio.pisani@unina.it

**Keywords:** chronic kidney disease, mineral bone disorder, erythropoietin, ESKD, Klotho, anemia

## Abstract

Anemia and mineral and bone disorder (MBD) are significant complications of chronic kidney disease (CKD). The erythropoietin (Epo) pathway plays a key role in both of these processes in CKD. Another molecule that plays an important role in CKD-MBD is fibroblast growth factor (FGF)-23, whose main role is to maintain serum phosphate levels in the normal range, acting via its co-receptor Klotho; however, its activity may also be related to anemia and inflammation. In this review, the regulation of Epo and FGF-23 and the molecular mechanisms of their action are outlined. Furthermore, the complex interaction between EPO and FGF-23 is discussed, as well as their association with other anemia-related factors and processes such as Klotho, vitamin D, and iron deficiency. Together, these may be part of a “kidney–bone marrow–bone axis” that promotes CKD-MBD.

## 1. Introduction

Erythropoietin (Epo) is considered the key protein in the complex proliferation and differentiation processes that lead to the production of mature erythrocytes. However, several studies have also described a multitude of paracrine and endocrine effects of Epo. Indeed, various cell types, including neurons, cardiomyocytes, and the endothelial and smooth muscle cells of vessels, can synthesize Epo and present specific Epo receptors on their membrane surfaces.

In chronic kidney disease (CKD), the Epo pathway appears to be involved not only in the onset of anemia but also in the regulation of CKD-mineral and bone disorder (CKD-MBD), a systemic disorder characterized by biochemical abnormalities, bone disorders, and vascular calcification. A key player in CKD-MBD is fibroblast growth factor-23 (FGF-23), one of the primary regulators of phosphate metabolism.

**Figure 1:**
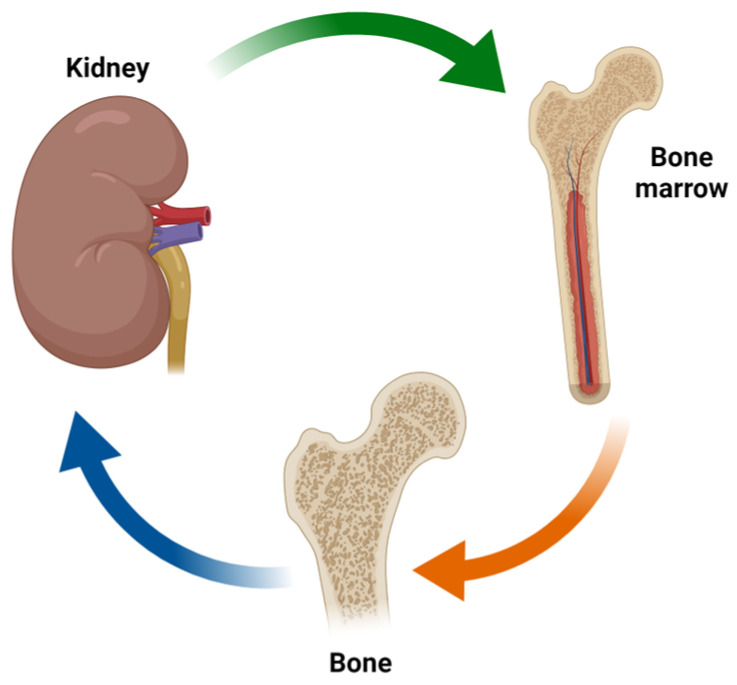
The kidney–bone marrow–bone axis in which complex hormonal systems are involved including FGF-23, Klotho, vitamin D, Epo, and several regulatory factors of iron metabolism.

This narrative review aims to describe the emerging complex relationships between anemia, anemia-related factors, FGF-23, and other relevant molecules implicated in the pathogenesis of CKD-MBD, defining a “kidney–bone marrow–bone axis” (Figure 1).

How FGF-23 and vitamin D play key roles in iron metabolism and the onset of anemia secondary to CKD will be elucidated in this paper. Feedback systems have been described between Epo and FGF-23—which is also produced in the bone marrow through the stimulation from Epo itself—similar to conditions of inflammation and iron deficiency. Klotho, a co-receptor of FGF-23, is also a key player in this “axis”, and its levels are reduced from the early stages of CKD and subsequently increase under Epo stimulation.

## 2. Factors Involved in the Kidney/Bone Marrow/Bone Axis

### 2.1. EPO: Synthesis, Regulation and Pathways

Human Epo, in its circulating form, is a glycoprotein hormone with a molecular mass of 30.4 KDa consisting of 165 amino acids to which four glycans are linked. In a developing fetus, Epo is generated by the liver. Meanwhile, in adulthood, the kidneys are the main sources of Epo, which is produced by the interstitial peritubular fibroblasts of the mid-cortical region. This region is characterized by high oxygen consumption and low oxygen supply, making hypoxia the major stimulus for Epo production. The endothelial cells of the peritubular capillaries act as oxygen tension sensors: depending on the oxygen level, more or fewer fibroblasts are activated to produce Epo [1,2].

The regulatory variable for the production of Epo is the tissue pO_2_, which is closely related to hemoglobin (Hb) concentration, arterial pO_2_, and Hb-O_2_ affinity. The kidneys are adapted for O_2_-dependent Epo production since pO_2_ in the renal cortical region is relatively unaffected by changes in blood flow [1,3].

The transcriptional regulation of the Epo gene (*EPO*), located on chromosome 7, is controlled by several transcription factors. The transcriptional activators responsible for the hypoxic induction of *EPO* are the heterodimeric transcription factors known as hypoxia-inducible transcription factors (HIFs) [4,5]. HIFs consist of an oxygen-sensitive α-subunit (of which there are three isoforms: HIF-1α, HIF-2α, and HIF-3α) and a constitutively expressed β-subunit, also known as ARNT (aryl hydrocarbon receptor nuclear translocator) [6,7,8,9].

Epo is primarily activated by HIF-2, consisting of the subunits HIF-1β and HIF-2α [10]. HIFs (HIF-1 and HIF-3) modulate gene expression by binding to specific DNA sequences called hypoxia response elements (HREs) [11].

The regulation of HIF activity relies predominantly on prolyl-hydroxylases (PHD), which are activated by blood levels of iron, oxygen, a-ketoglutarate, ascorbic acid (vitamin C), and reactive oxygen species (ROS) [12].

Another negative regulator of gene transcription is asparaginyl hydroxylase, also known as factor-inhibiting HIF (FIH), which hydroxylates HIF-1α and inhibits its interaction with cofactors [13]. Thus, in normoxia (where oxygen supply exceeds demand), HIFs are continuously degraded in an O_2_-dependent manner and cannot induce the transcription of target genes [12,14]. In hypoxic conditions, the activity of PHDs and FIH is reduced due to lower oxygen tension: HIF-1α is not degraded, allowing it to dimerize with HIF-1β. This complex translocates into the nucleus, activating hypoxia-induced response genes in the presence of CBP (CREB binding protein)/P300 [15]. This cascade results in the activation of *EPO*, and the subsequent increase in oxygen tension again leads to the degradation of HIF-1α.

Another important function of HIF is the regulation of transcription of genes involved in iron metabolism and oxygen transport. Specifically, HIF-1 promotes the transcription of genes encoding transferrin, ceruloplasmin (also known as ferroxidase), and transferrin receptor-1. HIF-2, which regulates HIF-1 activity, increases the synthesis of intestinal divalent cation transporters and ferroportin, thereby promoting iron uptake and transport to sites designated for erythropoiesis [16].

The Epo receptor (EpoR) is a transmembrane receptor belonging to the cytokine receptor superfamily, lacking kinase activity. It is localized on erythroid precursors and is also present, as mentioned above, in other nonhematopoietic tissues. Human endothelial, renal, cardiac, and neuronal cells contain 10- to 100-fold lower EpoR mRNA levels than those cells that are highly sensitive to Epo [17,18].

In the bone marrow, the cell types expressing the highest numbers of EpoRs are the colony-forming unit erythroid (CFU-E) and proerythroblasts. At the same time, EpoRs progressively decrease as the cells continue to differentiate toward the mature erythrocyte. Indeed, reticulocytes and mature erythrocytes lack EpoR [1].

The binding of Epo to its receptor results in homodimerization of the receptor [19], which is followed by the activation of a cytoplasmic tyrosine kinase from the Janus kinase (JAK) family, specifically JAK2 [20]. The phosphorylated EpoR triggers a sequence of intracellular cascades, thereby exerting anti-apoptotic, pro-differentiation, and proliferation effects. Indeed, Epo has been demonstrated to be protective in several in vitro or in vivo models in which detrimental substances/stimuli have been used to induce kidney cell damage/death [21,22,23,24,25,26]. Epo mRNA has also been found in other organs, such as in the bone marrow, spleen, liver, placenta, brain, reproductive tract, and lungs [1,3,27,28,29]. In these tissues, Epo is involved in non-erythropoietic functions.

Several pieces of evidence have shown that the brain can produce Epo (particularly in certain areas such as periventricular areas and from astrocytes) in response to hypoxia [30]. The expression in the central nervous system of *EPO* is thought to be crucial in normal brain development [31] and may play a role as a neuroprotector [32,33,34,35] and regulator of cerebral blood flow [36]. A common feature of brain and testicular *EPO* expression [28,37,38] is that its production at these sites is separated from the systemic circulation by the blood–brain barrier and blood–testicular barrier, respectively, suggesting more of a paracrine rather than endocrine action. Nonetheless, some evidence suggests that in certain conditions (such as those of brain damage) Epo may cross the blood–brain barrier [35].

Regarding the female reproductive tract, EPO expression in both the uterus [39] and oviduct was shown in rats [40], while in humans, it has been demonstrated to be present in the endometrium with variations related to the menstrual cycle [41]. Epo mRNA is also expressed in the placenta on both the fetal and maternal side [27,42,43]; its function here has not been determined, but it is thought to exert a trophic effect on the gastrointestinal tract [44]. Through high-sensitivity mRNA identification methods, EPO has also been found in the lungs, spleen, heart, and bone marrow [45]. In the latter site, it appears to be involved in erythropoiesis through an autocrine and/or paracrine mechanism but certainly with a significantly less-prominent role than circulating Epo. This is demonstrated by anemia of renal origin as a result of kidney failure [3]. EPO expression at the cardiac level is low but appears to be involved in actual cardiac morphogenesis [46].

### 2.2. CKD-MBD and Anemia: Different Patterns of Presentation

CKD-MBD is one of the most prognostically significant complications within the clinical spectrum of CKD. Calcium, phosphate, parathyroid hormone (PTH), FGF-23, and vitamin D metabolism, together with abnormalities in bone turnover, mineralization, volume linear growth, strength, and extra skeletal calcification, are involved in CKD-MBD [47,48,49].

The changes that are characteristic of CKD-MBD develop gradually as renal function declines, becoming clinically apparent when the estimated glomerular filtration rate (eGFR) falls below 40 mL/min/1.73 m^2^. Some of these elements manifest early in CKD, even before significant changes in plasma calcium, phosphate, PTH, and vitamin D levels are observed. Additionally, increased FGF-23 secretion, Klotho deficiency, decreased bone formation rates, and vascular calcification are present [50,51,52,53,54,55].

The spectrum of CKD-MBD differs in both the degree of CKD and the cause of kidney damage. Renal osteodystrophy is just one component of the bone abnormalities of CKD-MBD and defines changes in bone morphology from CKD evidenced by bone biopsy. Four main types of renal osteodystrophy are recognized: osteitis fibrosa (formally known as osteitis fibrosa cystica), osteomalacia, adynamic bone disease, and mixed disease [47].

Dynamic bone disease clearly prevails in patients with diabetic kidney disease upon conservative treatment [56,57,58], and lower levels of PTH and alkaline phosphatase have also been found in diabetic patients receiving dialysis compared with non-diabetics [59,60]. In this same population (diabetic dialysis patients), a higher prevalence of vertebral fractures has been demonstrated than in non-diabetics [56,57,58,59,60,61].

Even in patients with autosomal dominant polycystic kidney disease (ADPKD), lower levels of alkaline phosphatase and PTH together with higher circulating intact FGF-23 (iFGF-23) and decreased bone formation rate have been demonstrated [62,63]. However, ADPKD is not associated with higher rates of bone fracture in ESKD patients [63].

In patients with glomerular diseases, especially those with nephrotic syndrome, bone disorders have a very complex and varied etiopathogenesis due to the influence of vitamin D homeostasis (urinary loss of vitamin D-binding protein), the use of corticosteroids and other immunosuppressive drugs, the inflammatory environment, and the actions of immune cells and factors. In patients with nephrotic syndrome, a high occurrence of osteomalacia (associated or not with high bone turnover), likely secondary to reduced vitamin D concentrations, has been shown [64]. In this population, the contribution of glomerular disease to increased fracture risk has been poorly explored.

Even the degree of anemia varies based on the type of kidney disease, the stage of progression, and associated complications. Indeed, in patients with ADPKD, the level of anemia is typically lower than in other causes of CKD [65,66,67,68], and some of them have normal hemoglobin values. Epo levels are, on average, twice as high as in patients with ESKD from other causes [69]. This is probably related to the progressive expansion of cysts that results in the compression of blood vessels leading to regional hypoxia and up-regulation of HIF [70]. The HIF-2α-mediated stimulation of Epo production may explain why ADPKD patients present with less severe anemia than other patients with CKD [69].

### 2.3. FGF-23: Production and Main Actions

**Figure 2:**
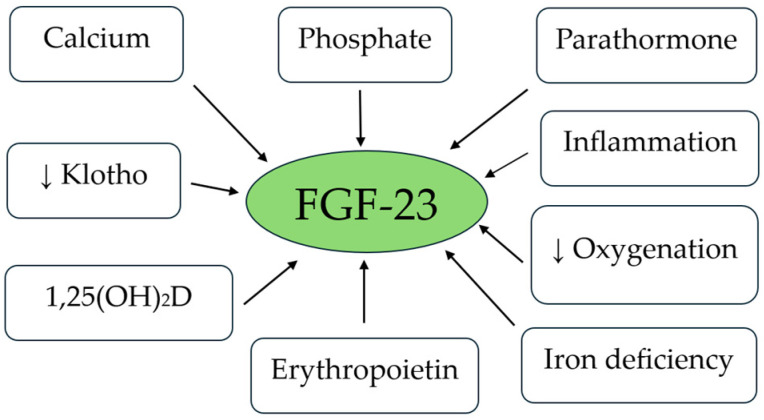
Factors stimulating FGF-23 production. “↓” means a reduction in levels.

FGF-23 is a hormone produced by osteocytes and osteoblasts in bone stimulated by increased dietary phosphate load, calcitriol, PTH, and calcium (Figure 2). FGF-23 acts on the kidney, parathyroid, heart, bone, and potentially other organs [71,72,73,74,75,76,77,78,79,80].

FGF-23, along with FGF-19 and FGF-21, belongs to a subfamily of endocrine FGFs [81]. FGFs signal through four FGF tyrosine kinase receptors (FGFR1–4), activating the RAS-MAPK and PI3K-AKT pathways [82]. Classical FGF-23 signalling requires the co-receptor α-Klotho, a transmembrane protein with extracellular glucuronidase activity [83], to bind to the FGF receptor 1c (FGFR1c) [84]. Additionally, FGF-23 can act independently of Klotho by activating FGFR3 and FGFR4 [80,85].

**Figure 3:**
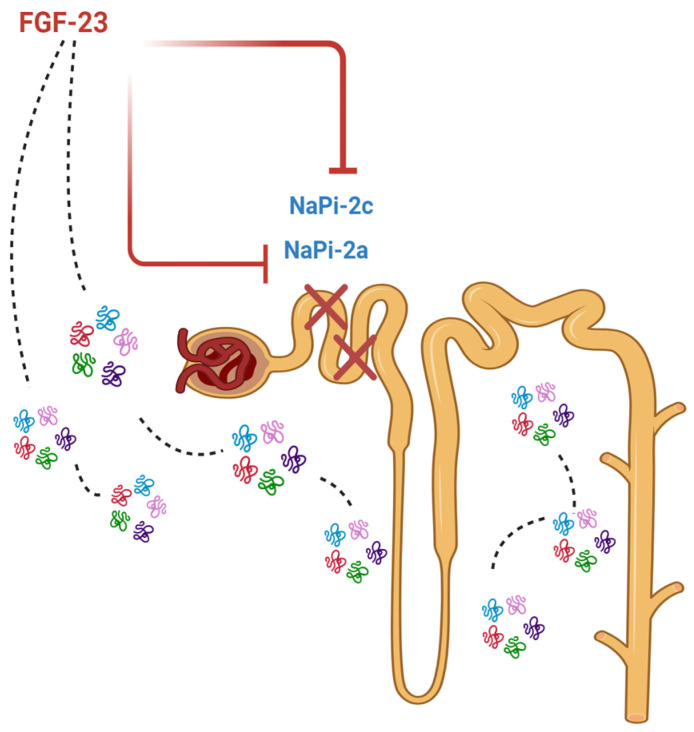
FGF-23, by binding to its own receptor and to the Klotho coreceptor, induces the downregulation of the cotransporter Na/Pi IIa and Na/Pi IIc transporters with a consequent phosphaturic effect.

The main action of FGF-23 is to maintain serum phosphate concentration in the normal range by reducing its renal and intestinal absorption, primarily through the suppression of calcitriol production [86]. FGF-23 is a key homeostatic regulator of phosphate and acts as a counterregulatory hormone to 1,25-dihydroxyvitamin D (1,25(OH)_2_D) (or calcitriol). The phosphaturic effects of FGF-23 in the kidney tubules are Klotho dependent. In renal proximal tubular cells, FGF-23 binds to FGFR and its coreceptor, Klotho, leading to downregulation of the luminal membrane sodium phosphate cotransporter Na/Pi IIa (and possibly the Na/Pi IIc transporter) [87,88] (Figure 3).

Additionally, FGF-23 inhibits the expression of the enzyme alpha-1 hydroxylase in the proximal tubule, which converts 25(OH)D to active 1,25(OH)_2_D [89] and increases expression of renal 24-hydroxylase, which converts 25(OH)D and 1,25(OH)_2_D to inactive metabolites, thereby reducing renal production of calcitriol [90,91,92]. Consequently, these actions ultimately reduce serum phosphate concentration.

Increased FGF-23 is an early biomarker of CKD-MBD identifiable before any changes in serum levels of calcium, phosphate, and PTH [54,55]. FGF-23 levels begin to increase five years before the onset of ESKD and continue to increase rapidly until the transition to ESKD [55]. Although the progressive rise in FGF-23 helps maintain normophosphataemia until the later stages of CKD [54,76,93,94], it leads to the suppression of calcitriol production, promoting secondary hyperparathyroidism.

Other direct stimulators of FGF-23 include Lipocalin 2 (LCN2, a marker of inflammation and an iron transporter) [95], reduced oxygenation, iron deficiency, and Epo [96] (Figure 2).

Several clinical studies on CKD patients have shown that increased FGF-23 was associated with poor patient outcomes [97,98,99,100]. Furthermore, Klotho deficiency did not confound the association between FGF-23 and mortality or heart failure hospitalization [101].

An increasing correlation between the mechanisms regulating calcium-phosphate metabolism and erythropoiesis is evident from recent scientific literature. As described later, FGF-23 promotes anemia and systemic inflammation.

### 2.4. Vitamin D and Anemia

In patients with CKD, vitamin D deficiency accelerates the development of MBD with reduced bone mineral density [102,103,104] and is associated with several adverse clinical outcomes, such as anemia [105,106,107], cardiovascular events [108], and increased mortality [108,109].

In vitro studies have demonstrated the presence of the vitamin D receptor (VDR) on the cell membrane of macrophages. Activation of VDR inhibits the synthesis of proinflammatory cytokines and stimulates the release of anti-inflammatory cytokines, promoting the proliferation of erythroid precursors. Therefore, vitamin D deficiency in CKD could be a key factor in maintaining a proinflammatory state, leading to ineffective erythropoiesis. Using vitamin D (both native and active forms) and paracalcitol (a selective VDR activator) may help reduce inflammation and improve anemia in CKD subjects, potentially lowering the doses of Epo required to achieve the same effect on erythropoiesis [110].

Many clinical studies suggest that vitamin D supplementation may improve the response to erythropoiesis-stimulating agents (ESAs) [111]. These results may be attributed to the immunomodulatory and hepcidin-reducing properties of vitamin D. For instance, a study on healthy individuals showed that a single high dose of cholecalciferol significantly reduced serum hepcidin levels [112]. Hepcidin, an acute phase protein, reduces iron availability by promoting iron sequestration [113]. Furthermore, activation of VDR-related transcription factors can enhance the growth of erythroid progenitors [114].

Conversely, a randomized controlled trial showed that cholecalciferol supplementation increased serum hepcidin levels on Day 3 among ESKD patients. However, it also increased serum 1,25(OH)_2_D levels and decreased the dose of active vitamin D drugs needed [115]. This indicates a complex relationship between vitamin D supplementation and hepcidin regulation in CKD patients, suggesting the need for further research to optimize treatment strategies.

### 2.5. FGF-23 and Iron Deficiency

In CKD patients, iron deficiency does not lead to a decrease in absolute blood iron levels but rather to a failure in utilizing iron for erythropoiesis, a condition known as functional iron deficiency. This is primarily due to the chronic inflammatory state that characterizes CKD, which hampers iron utilization for erythropoiesis.

Initial observations on patients with autosomal dominant hypophosphatemic rickets (ADHR) suggested that iron deficiency might regulate FGF-23 synthesis. ADHR is characterized by elevated FGF-23 levels, causing hypophosphatemia and osteomalacia/rickets [116]. Female ADHR patients show hypophosphatemic flares coinciding with menses and pregnancy, conditions associated with iron deficiency, suggesting increased FGF-23 production [116].

A study of elderly men with CKD in Sweden showed that low iron levels were associated with high serum FGF-23 levels independent of renal function and inflammation [117]. This correlation was also described in a large prospective study involving CKD patients [118] and kidney transplant recipients (KTRs) [119].

Iron deficiency upregulates furin, a proprotein convertase that cleaves iFGF-23, by stabilizing HIF1-α, thereby leading to cleavage of FGF-23 into C-terminal cleavage fragments (c-FGF-23) [120,121]. Thus, iron deficiency not only increases FGF-23 production but also stimulates its cleavage into fragments [122]. The role and function of these cleaved fragments in CKD patients are still not well understood. Nonetheless, iFGF-23 is considered the bioactive form, and its high levels are associated with poor prognosis in CKD [123].

In CKD, some factors may increase FGF-23 transcription, leading to higher levels of the intact form of FGF-23. One of these conditions, iron deficiency, can be corrected to reduce FGF-23 production. Therefore, iron supplementation may offer therapeutic benefits in managing FGF-23 levels. A prospective randomized study evaluated oral and intravenous (IV) iron use in hemodialysis patients with iron-deficiency anemia. The results found that serum cFGF-23 levels decreased in both groups. However, serum iFGF-23 levels increased in the IV iron group, indicating that oral iron might be more effective in preventing increased iFGF-23 expression [124].

Treatments with iron-based phosphate binders, such as sucroferric oxyhydroxide and ferric citrate, have been shown to reduce serum FGF-23 levels. Sucroferric oxyhydroxide notably reduced serum FGF-23 levels in hemodialysis patients [125], while ferric citrate also decreased serum phosphate levels and improved iron parameters, lowering serum FGF-23 levels [126].

Similarly, oral iron supplementation in ADHR patients reduced the high FGF-23 levels [127], whereas certain IV iron preparations, such as those with carboxymaltose backbones, increased iFGF-23 levels [128].

In mouse CKD models, improving iron utilization with Epo and HIF-PHD inhibitors significantly reduced iFGF-23 levels, demonstrating that iron management in CKD patients could improve mineral metabolism outcomes [129].

### 2.6. FGF-23 and Epo

Renal anemia is caused by several factors, including reduced Epo production, bone marrow suppression, reduced erythrocyte survival, impaired iron metabolism, and chronic inflammation [130].

Anemia [131,132] significantly affects daily activities, cardiovascular health, and overall prognosis in CKD patients [133,134,135]. The development of recombinant human Epo (rhEpo) over 30 years ago revolutionized the treatment of anemia in CKD [136]. The interaction of rhEpo on serum FGF-23 levels is complex and multifaceted. The role of Epo as a physiological regulator of FGF-23 production was investigated in a mouse model of acute hemorrhage. A severe and acute reduction in blood volume, leading to tissue ischemia, stimulated Epo synthesis. It resulted in the proliferation of erythroid cells in the bone marrow and the release of immature erythrocyte precursors into circulation. In these mice, an increase in Epo levels accompanied by an increase in plasma levels of the C-terminal fragment of FGF-23 was observed within 6 h of the hemorrhagic event [137].

In a recent study, Daryadel et al. observed that administering rhEpo to healthy mice resulted in an acute increase, over a 24-h period, in the concentration of the C-terminal fragment of FGF-23 and, after a few days, an increase in iFGF-23. This acute increase in FGF-23 did not, as might be expected, lead to a reduction in serum phosphates or PTH levels. FGF-23 mRNA expression was evident in the hematopoietic marrow, particularly in erythroid pre-cursors, but not in osteoblasts and osteocytes. Administration of recombinant FGF-23 in the same mice, however, reduced *EPO* transcript expression at the renal level, likely due to a negative feedback mechanism [138].

This direct relationship between FGF-23 and Epo has already been reported. In wild-type mice, administering recombinant human FGF-23 decreased serum Epo levels [139], and the administration of FGF-23-blocking peptide increased serum Epo levels [140]. These observations suggest that FGF-23 suppresses erythropoiesis in mice.

However, in mice with CKD, rhEpo injections resulted in elevated circulating total FGF-23, more than levels of iFGF-23, suggesting the coupling of increased FGF-23 production with increased proteolytic cleavage [141]. In these murine models, the bone marrow was shown to be a novel source of EPO-stimulated FGF-23 production. In humans, serum Epo levels and rhEpo dose are positively and independently associated with total FGF-23 (but not iFGF-23) levels across the spectrum of CKD and after kidney transplantation. These data were consistent with the effects of Epo on FGF-23 production and metabolism observed in the murine models [141]. Similar data were observed in patients with both CKD and chronic heart failure, where exogenous EPO injections markedly increased cFGF-23 expression [123,142].

Therefore, similar to inflammation and iron deficiency, Epo increases both *FGF-23* transcription and FGF-23 cleavage. Notably, Epo induces non-osseous *FGF-23* mRNA expression in the bone marrow [137,138,141,143,144,145].

This finding could support the hypothesis that elevated FGF-23 levels, as observed in subjects with CKD, may contribute, along with other factors, to reduced Epo production.

The mechanisms by which Epo increases bone marrow and bone transcription of FGF-23 are not known, nor are the reasons why Epo increases post-translational cleavage of FGF-23. The regulation of post-translational cleavage of FGF-23 is complex and involves several enzymes, including GALNT3 (N-acetylgalactosaminyltransferase), FAM20C (family with sequence similarity 20, member C), Furin, and PC5/6 (Pcsk5) [146,147].

### 2.7. FGF-23 and ESA Hyporesponsiveness

One of the prominent issues in managing anemia in CKD patients, especially those undergoing dialysis treatment, is resistance to ESAs. This condition has multiple causative factors, including chronic inflammation. Circulating proinflammatory cytokines reduce or sometimes suppress the proliferation of erythroid precursors and stimulate the synthesis of hepcidin, a liver-derived hormone [148]. Hepcidin results in iron sequestration in bone marrow macrophages, reducing its bioavailability for erythropoiesis and inhibiting enteral iron absorption [149].

A positive association between FGF-23 levels and markers of inflammation has been clearly demonstrated in cohorts of humans [150,151]. In in vitro studies with osteocyte-like cell lines, pro-inflammatory cytokines have been seen to cause the upregulation of *FGF-23* mRNA expression [152]. In vivo, inflammatory stimuli have been shown to increase in both *FGF-23* transcription and FGF-23 cleavage [153]. In mice, the exogenous administration of hepcidin resulted in an acute increase in the bone expression of *FGF-23*, with an increase in the total circulating levels of FGF-23 and maintenance of iFGF-23 within normal ranges [153].

In a large study that included 1044 hemodialysis patients from the Japanese Dialysis Outcomes and Practice Pattern Study, it was found that the lowest and highest levels of FGF-23 were associated with ESA hyporesponsiveness [154]. The finding that ESA hyporesponsiveness was present in patients with low FGF-23 levels was novel and did not support the idea that only high FGF-23 promotes ESA resistance [118,155]. Low FGF-23 levels may suggest limited FGF-23 production due to malnutrition, a condition in which an energy-saving mechanism might operate in bone marrow and osteocytes. Malnutrition–inflammation complex syndrome is associated with hyporesponsiveness to ESA in hemodialysis patients [156].

Another factor contributing to ESA resistance is secondary hyperparathyroidism, characterized by increased synthesis and secretion of PTH resulting from hyperphosphatemia and hypocalcaemia. Although the increase in blood levels of PTH is an adaptive mechanism the body adopts to maintain proper calcium and phosphate homeostasis, continuous bone remodelling by osteoclasts, in the long term, results in bone marrow fibrosis and a consequent reduction in erythropoiesis [157].

Thus, correcting secondary hyperparathyroidism arises as a therapeutic strategy to improve anaemia in CKD subjects. Some studies evaluated the effect of calcimimetic therapy in patients undergoing maintenance hemodialysis. The administration of cinacalcet resulted in an increase in hemoglobin levels in treated patients and a lower demand for ESAs [158,159,160,161].

Treatment of secondary hyperparathyroidism with cinacalcet not only improved anemia in HD patients [162] but was also associated with reduced FGF-23 levels [163].

### 2.8. FGF-23 and HIFs

As previously described, HIFs are proteins that regulate transcription in response to low cellular oxygen levels. PHD regulates HIFs in an oxygen-dependent manner [164]. Hypoxia-inducible factor prolyl hydroxylase inhibitors (HIF-PHI) are compounds developed for the treatment of renal-derived anaemia through a novel mechanism of action. HIF-PHIs promote endogenous Epo production and improve iron supply for hematopoiesis [2,165,166,167].

Roxadustat, an HIF-PHI compound, increases FGF-23 production, suggesting that HIF-PHI could influence the expression and cleavage of FGF-23 through the induction of endogenous *EPO* transcription [138,153]. Another study reported that use of HIF-PHI increases FGF-23 expression [153].

Several studies have identified the molecular mechanisms involved, showing that inflammation or iron deficiency induces HIF-1α [153] and that the binding of HIF-1α increases its synthesis in osteogenic cells [168]. Additionally, HIF-1α could indirectly increase the expression of FGF-23 through the induction of endogenous Epo production, promoting FGF-23 transcription and cleavage [137,141,143,144,169]. It is worth noting that following treatment with HIF-PHI, most of the induced FGF-23 is cFGF-23 [144,153].

Contrary to this hypothesis, a recent study on a cohort of hemodialysis patients undergoing a switch from Darbepoetin-α to Roxadustat showed no significant increases in Epo levels after switching to Roxadustat, but erythroferrone levels were continuously elevated, like darbepoetin-α treatment [170]. Instead, hepcidin-25 and total iron binding capacity were significantly decreased or increased in the group of patients undergoing Roxadustat therapy [170].

Levels of iFGF-23 remain normal, while those of total FGF-23 increase due to enhanced cleavage of FGF-23 [120]. The pathogenetic significance of the increased expression of c-FGF-23 remains unknown, and future studies are needed to elucidate the biological activities of the C-terminal fragments of FGF-23.

### 2.9. Klotho and Anemia

The effects on the kidney of FGF-23 depend on the presence of the Klotho protein, which is expressed in the distal tubule and other organs, such as the parathyroid glands.

The extracellular portion of the transmembrane form of Klotho (tm-Klotho) is cleaved by some metalloproteases, forming the soluble form (s-Klotho), which has paracrine effects like those of FGF-23 but independent of its synthesis. From the earliest stages of CKD, a reduced expression of tm-Klotho has been observed, and this also results in reduced production of s-Klotho.

In a mouse model of nephropathy, Sugiura et al. observed that mRNA expression and synthesis of circulating Klotho protein were significantly reduced, resulting in increased serum phosphates. Administration of rHuEpo in these mice resulted in reduced phosphoremia [171].

In a study of 117 kidney transplant recipients (KTRs) receiving rHuEpo therapy and 22 healthy subjects, Leone et al. showed that s-Klotho levels were significantly higher in KTRs than in controls. Additionally, in KTRs these levels correlated proportionally with eGFR and FGF-23 levels. In 17 KTRs, rHuEpo therapy was discontinued for 5 weeks, and resulted in a marked reduction in s-Klotho levels compared with patients who continued therapy. The same group also evaluated Klotho mRNA expression and related protein synthesis in HK-2 tubular cells treated with cyclosporine and rHuEpo alone or in combination. Cells treated first with rHuEpo and then with cyclosporine showed higher Klotho mRNA expression than those treated with one drug alone [172].

These studies have shown that EPO promotes not only FGF-23 but also Klotho synthesis.

A study by Sari et al. demonstrated a significant increase in FGF-23, s-Klotho, and PTH levels in a cohort of ADPKD patients compared with healthy subjects. By contrast, in subjects with CKD of different etiologies, s-Klotho levels are generally reduced. ADPKD patients have higher mean hemoglobin values than CKD patients, likely due to hyperactivation of the transcription factor HIF, secondary to the hypoxic state that occurs due to the compression of cysts on the renal vascular bed. Increased HIF activity would result in increased Epo synthesis, which could account for the increased Klotho synthesis observed in previous studies [173].

## 3. New Biomarkers of Mineral Bone Disease

To identify the type (and degree) of renal bone disease, the gold standard remains bone biopsy because no combination of laboratory markers has so far proven sufficiently accurate. Calcium, phosphorus, vitamin D, PTH, and the bone-specific alkaline phosphatase isoenzyme (BSAP) are the main biomarkers used to assess renal bone disease. Other biomarkers of bone turnover used in the evaluation of osteoporosis are not as useful in the assessment and management of CKD-MBD. Indeed, serum C-telopeptide (CTX) and monomeric forms of serum propeptide type I collagen (PINP), respectively the most useful markers of bone resorption and formation, are cleared by kidney [174,175,176]. There are other biomarkers of bone turnover that are not excreted by the kidney, and they may have a better predictive capacity for fracture risk in patients with CKD not on dialysis. They include BSAP, tartrate resistant acid phosphatase (TRAP5b, an osteoclast cellular marker), and the trimer form of PINP [175,177,178].

In recent years, impaired Wnt–β-catenin signaling has emerged as playing a potential role in the pathogenesis of CKD-MBD [179]. Sclerostin and Dickkopf-1 (Dkk-1) are two soluble inhibitors of Wnt signaling [180]: sclerostin (produced by osteocytes [181]) is expressed at the sites of bone formation in bone and cartilage [182], whereas Dkk-1 (produced by osteoblasts and osteocytes) is expressed in many tissues during embryogenesis, including bone [183]. Sclerostin acts on pre-osteoblastic and osteoblastic cells, leading to a reduction in osteoblastic proliferative and differentiative activity [181]. In the course of CKD, circulating levels of Sclerostin increase proportionally to the reduction in glomerular filtrate [184] due to enhanced bone production [52]. Changes in Sclerostin synthesis during CKD are early and appear to develop before alterations in PTH or FGF-23 [185] and play a role in influencing bone turnover. In hemodialysis patients, Sclerostin levels show a positive correlation with the development of a dynamic bone disease independent of PTH values against which a Sclerostin-mediated bone resistance seems to develop [186]. Sclerostin appears to be a promising indicator (especially in dialysis patients) of high turnover along with PTH, while Dkk1 has not been shown to correlate with histomorphometric bone parameters [179,186]. It had previously been shown that Sclerostin concentration was higher in hemodialysis patients and that its levels were negatively correlated with those of PTH [187]. Results from clinical trials are not unambiguous regarding the association between Sclerostin levels and cardiovascular morbidity/mortality in patients with CKD. Indeed, in hemodialysis patients, while data from small populations have found a correlation between Sclerostin levels and mortality [188,189,190], data from a larger population (NESOCAD study) showed, in contrast, reduced survival of patients with the lowest Sclerostin levels [191].

Also, levels of osteoprotegerin (a secreted soluble receptor produced by various cells including osteoblasts) could be an effective and early serum biomarker for the diagnosis of CKD-MBD [192,193], and its levels appear to be associated with increased mortality (both in patients with CKD on conservative and dialysis treatment) [194].

Fetuin-A is a hepatocyte-derived protein (hepatokine) with multifaceted functions [195], among them inhibiting extraosseous calcifications, transport protein for calcium and phosphate in colloidal calciprotein particles, and involvement in bone mineralization [196]. Its role in vascular calcification is not yet fully elucidated, but low levels of Fetuin-A correlate with aortic calcifications and major clinical events [197,198]. In patients with CKD, serum levels of Fetuin-A are significantly reduced [199,200], and several studies have shown that, in this population, reduced circulating levels of Fetuin-A are associated with increased all-cause and cardiovascular mortality [201,202].

In recent years, knowledge about the complex mechanisms regulating mineral metabolism has brought to our attention new molecules involved in these processes. Among those examined, however, we can attribute the biomarker that is characteristic of CKD-MBD mainly to FGF-23. As for the previously described molecules (and others as well), their roles in bone and/or cardiovascular involvement still remain to be better defined, especially with regards to refinement of the assay method in biological fluids.

## 4. Discussion

**Figure 4:**
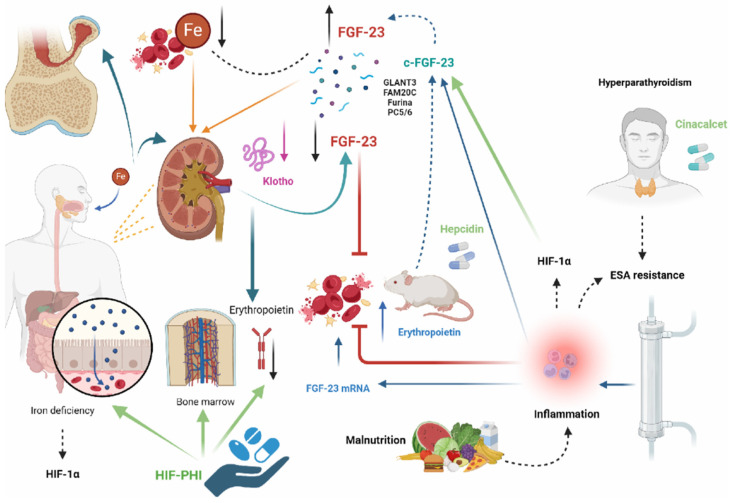
The main actions of FGF-23 and major therapeutic implications in the management of anemia due to CKD and secondary hyperparathyroidism.

In this narrative review, we have analysed the main evidence from in vitro, in vivo, and human/clinical studies regarding the relationships between anemia secondary to CKD and related mineral and bone disease. CKD-MBD is one of the most prognostically significant phenomena of CKD, with elevated mortality rates primarily due to cardiovascular complications [203]. In this context, one of the key actors is FGF-23, a hormone whose primary role is to maintain serum phosphorus concentration within certain limits through reduced renal and intestinal absorption [86]. Its levels begin to rise very early in CKD, before the alterations in calcium, phosphorus, and PTH levels [54,55], and, in addition, its production is stimulated by reduced oxygenation, iron deficiency, and Epo [96]. FGF-23 promotes anemia and systemic inflammation and has been associated with poor outcomes in CKD patients [97,98,99,100]. Iron deficiency, one of the factors mainly responsible for anemia in CKD, has been found to be associated with elevated FGF-23 levels in the elderly with CKD [117,118] and in kidney transplant patients [119]. Therefore, correction of iron deficiency was associated with reduced FGF-23 production (Figure 4). In some studies, in hemodialysis patients, oral iron supplementation has been found to be associated with reduced levels of both cFGF-23 and iFGF-23 [124,125,126], demonstrating that proper iron management can also result in positive effects in mineral metabolism outcomes.

The connections between FGF-23 and rhEpo are very complex and varied, and the role of Epo as a regulator of FGF-23 production was first investigated in a mouse model of acute hemorrhage. Specifically, 6 h after the bleeding event, increased Epo levels were accompanied by increased plasma levels of cFGF-23 [129]. Recently, however, it has been reported that administration of rhEpo in healthy mice resulted in an acute increase in cFGF-23 and, after a few days, an increase in iFGF-23, without causing a reduction in serum phosphate and PTH levels. This was attributed to FGF-23 production mainly occurring in hematopoietic cells and not in osteoblasts and osteocytes. In addition, administration of recombinant FGF-23 in the same mice led to reduced EPO expression, demonstrating the existence of a negative feedback mechanism [138]. In mice with CKD, rhEpo administration resulted in increased circulating levels of total FGF-23, more than those of iFGF-23, suggesting an association between increased FGF-23 production and increased proteolytic cleavage [141]. These data demonstrated a novel source of EPO-mediated FGF-23 production taking place in the bone marrow. Similar results were also observed in humans, in whom Epo levels and rhEpo doses were positively and independently associated with total FGF-23 levels (but not iFGF-23) [123,142]. Therefore, similar to inflammation and iron deficiency, Epo increases both FGF-23 transcription and FGF-23 cleavage. Notably, Epo induces non-osseous FGF-23 mRNA expression in the bone marrow [137,138,141,143,144,145] (Figure 4).

These results support the hypothesis that the high levels of FGF-23 present in subjects with CKD may contribute, undoubtedly together with other factors, to the reduced production of Epo and consequently to the onset of anemia.

Another determining factor for anemia is certainly represented by hyporesponsiveness to ESA. Indeed, an association between FGF-23 levels and some markers of inflammation (a known contributor to the reduced response to ESA) has been found in a human cohort [150,151]. Surprisingly, it was shown in a large population of hemodialysis patients that both higher and lower FGF-23 levels were associated with hyporesponsiveness to ESA [154], which contradicts the idea that only high FGF-23 levels determine ESA resistance [118,155]. Another element that is believed to contribute to ESA resistance is secondary hyperparathyroidism since the bone remodulation induced by osteoclasts can result in bone marrow fibrosis and, consecutively, in reduced erythropoiesis [157]. It was seen that treatment of secondary hyperparathyroidism by administration of cinacalcet resulted in increased hemoglobin levels [158,159,160,161,162] and was also associated with reduced FGF-23 levels [163] (Figure 4).

However, the data from studies on HIF-PHI and FGF-23 are contrasting. Initially, Roxadustat was associated with an increase in FGF-23 production (probably through EPO transcription), while recently it was observed that in patients on hemodialysis, switching from Darbepoetin to Roxadustat did not result in an increase in Epo levels [170] (Figure 4).

## 5. Conclusions

FGF-23 is associated with CKD-MBD and anemia, suggesting that it could be the connecting factor between these conditions. The regulation of FGF-23 is very complex and heterogeneous, occurring at both transcriptional and post-transcriptional levels, involving bone, mineral, and renal factors. Non-mineral factors, such as iron deficiency, Epo, and inflammation, affect FGF-23 production and metabolism.

## 6. Future Directions

Further research is needed to elucidate how these factors increase FGF-23 transcription and to understand the physiological and pathological implications of elevated FGF-23 fragment levels. Understanding these mechanisms could provide new insights into managing CKD-MBD and anemia, potentially improving patient outcomes.
